# Studying the freely-behaving brain with fMRI

**DOI:** 10.1016/j.neuroimage.2012.01.009

**Published:** 2012-08-15

**Authors:** Eleanor A. Maguire

**Affiliations:** Wellcome Trust Centre for Neuroimaging, Institute of Neurology, University College London, 12 Queen Square, London WC1N 3BG, UK

**Keywords:** Naturalistic, Ecological, Virtual reality, Navigation, Autobiographical memory, fMRI, MVPA, Hippocampus

## Abstract

Given that the brain evolved to function in the real world then it seems reasonable to want to examine how it operates in that context. But of course the world is complex, as are the brain's responses to it, and MRI scanners are inherently restrictive environments. This combination of challenges makes the prospect of studying the freely-behaving brain with fMRI disconcerting to anyone sensible. When designing naturalistic fMRI experiments it is necessary to ensure that the thoughts or behaviours under scrutiny are not unduly perturbed or constrained by the imaging process, while still being amenable to experimental manipulation and control, and result in meaningful and interpretable data. This is difficult to achieve. Here, briefly, and in a highly subjective and selective manner, I consider: why we might want to deploy free-behaviour designs in an fMRI context, how to go about it, review some examples of it in action, and decide finally whether it is worth it (it is).

## Why do it?

Back in the mid-late 1990's, we and others had already conducted some PET studies of the freely-behaving brain (e.g. navigation in large-scale environments, recollection of autobiographical memories), and were making the move into fMRI (Aguirre et al., 1996; Ghaem et al., 1997; Grön et al., 2000; Maguire et al., 1996, 1997, 1998a,b; Roland and Friberg, 1985; Roland et al., 1987). Despite sensible results, studies such as these remained niche, and instead the main focus in the field was on conducting experiments with simple, easy to control and manipulate stimuli (e.g. word pairs) derived from the experimental and cognitive psychology traditions. In fact, studying the freely-behaving brain was explicitly frowned upon in some quarters. I was a post-doc with Chris Frith at the time, and was dispatched to meet and greet an eminent imaging speaker visiting our unit. During the conversation that ensued, he said “Why on earth are you wasting your time doing such uncontrolled experiments?” He explained that nothing useful could come of such an endeavour, that I was just adding layers of unnecessary complexity, resulting in essentially uninterpretable data. This, not uncommon, view, combined with the distain of some electrophysiologists for the value of functional neuroimaging studies of memory, meant these were occasionally disheartening times.

But thanks to the encouragement of Chris Frith, the methodological insights of Karl Friston, and the enthusiastic ‘can do’ attitude of Richard Frackowiak, we persevered; and for good reason. While fMRI experiments involving systematic manipulations of simple, static, controlled stimuli are undoubtedly desirable and appropriate for addressing many research questions, there are some situations where this approach falters. If one wants to understand how the brain perceives the continuous and complex multi-modal inputs it receives (Bartels and Zeki, 2004; Hasson et al., 2004), and parses them into meaningful events (Moran et al., 2004; Zacks et al., 2001) then simple, static stimuli simply will not do. Likewise, to appreciate how the brain interacts with the environment, learns and navigates a spatial layout through direct experience, then dynamic naturalistic stimuli are clearly required (Burgess et al., 2002; Maguire, 2007; Maguire et al., 1999; Spiers and Maguire, 2007a). Similarly, having people learn and recall single or sets of items certainly affords insights into memory processes, but does not capture the personally-experienced, rich, vivid, and often dynamic nature of autobiographical memories (Cabeza and St Jacques, 2007; Gilboa, 2004; Maguire, 2001; McDermott et al., 2009; Svoboda et al., 2006). In particular, without recourse to the free recall of people's unique and complex personal experiences over a lifetime, it is not possible to examine the neural basis of remote memories in a convincing manner.

Studying how people engage in other critical functions such as imagining and planning for the future (Hassabis and Maguire, 2007, 2009; Hassabis et al., 2007a; Schacter and Addis, 2007), social interactions (Frith, 2007; Frith and Frith, 2006; Frith and Singer, 2008) and also more practical behaviours such as driving a vehicle (Calhoun and Pearlson, 2012; Spiers and Maguire, 2007b) further emphasise the need for free-behaviour designs in fMRI. Moreover, it is vital to verify whether results obtained in experiments that use simplified stimuli actually hold true under natural conditions, especially as findings are typically assumed to generalise. For example, functional specializations observed in traditionally-designed fMRI experiments might not be found in real-world contexts (or indeed vice versa), with potentially important theoretical implications. The point is that we need both constrained and free-behaviour designs in fMRI, although the latter remain in the minority. This is not surprising, given that fMRI experiments that seek to capture the freely-behaving brain are difficult to execute effectively.

## How to do it?

Having established that one's research question cannot be addressed using a standard protocol, then a number of issues need to be considered (see Spiers and Maguire, 2007a for more details), including the stimuli to be employed, the task design, and the nature of the data analysis. Mental simulation is one obvious source of naturalistic stimuli, be that imagining navigation (Kumaran and Maguire, 2005), recollecting autobiographical memories (Maguire, 2001; Svoboda et al., 2006), or thinking about the future (Schacter and Addis, 2007). These internally-generated experiences are the most challenging to control, manipulate and verify. The other key type of stimuli are those that are externally-generated and under the control of the experimenter to a greater degree. For instance, movies have been employed in order to understand how participants parse continuous and complex stimulation into more manageable and meaningful events (Zacks et al., 2001), and to examine the response profiles of functionally specialised regions during dynamic and naturalistic viewing (Hasson et al., 2004, 2010; Bartels and Zeki, 2004).

**Fig. 1:**
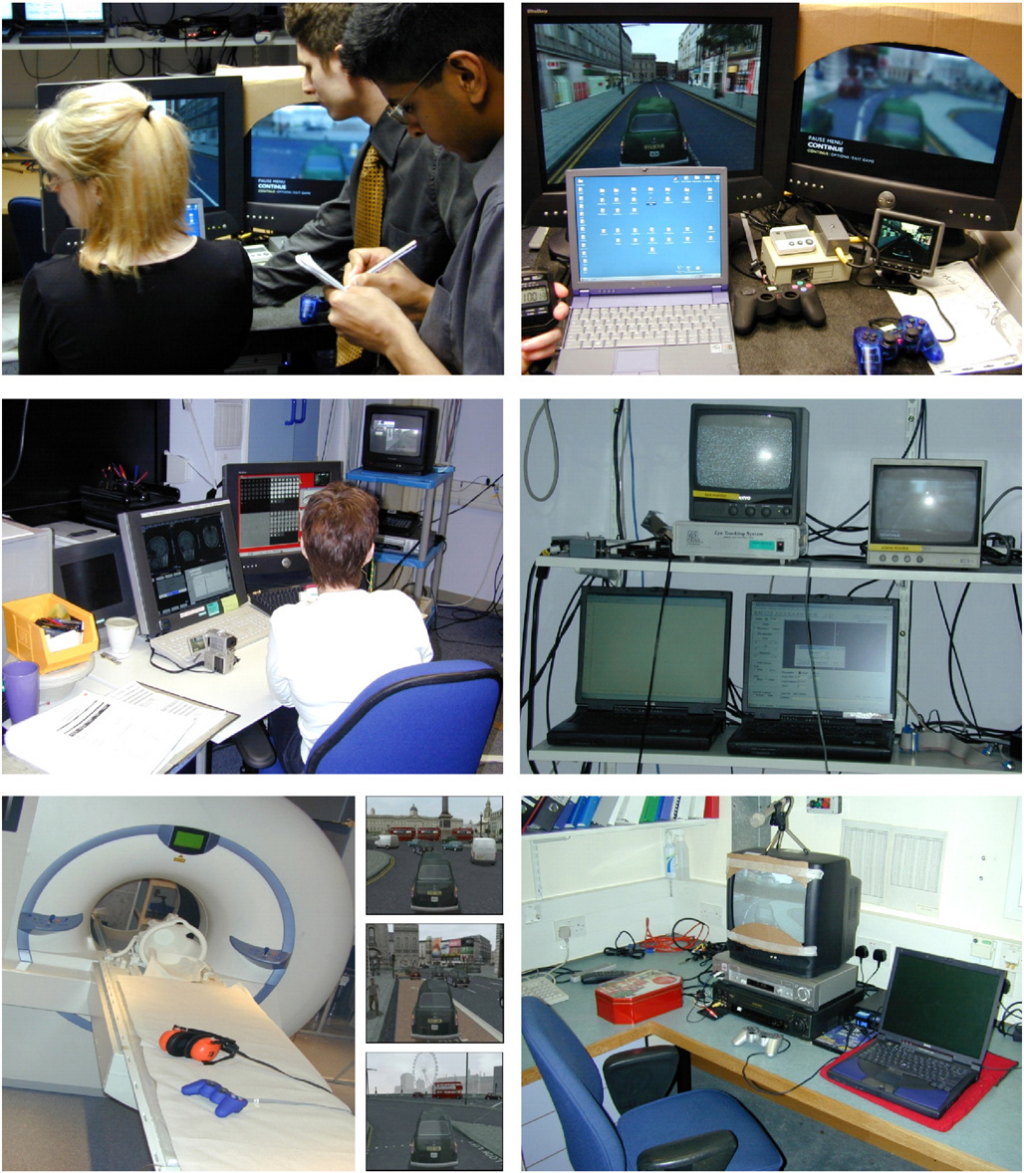
The complex set-up used to run a virtual reality navigation fMRI study (Spiers and Maguire, 2006a –— see text for details). This ‘behind the scenes’ view is rarely depicted in fMRI papers, and is in contrast to the experience of the participant (lower left) who uses a simple MRI-compatible games console, headphones and a screen to navigate in VR London. Snapshots of the simulation of London – middle bottom – are reproduced with the kind permission of Sony Computer Entertainment Europe.

One particularly significant advance that facilitated ecological fMRI experiments was the explosion in computer simulation technology in the eighties and nineties. Commercially-available video games are dynamic and interactive, with a first-person ground level perspective, and can have complex and naturalistic large-scale environments as their backdrops. These virtual reality (VR) games are sometimes accompanied by editors, allowing experimenters to manipulate (and record) aspects of the game to produce environments and scenarios suitable to address experimental questions. While such games offer exciting opportunities for real-world research in the scanner, their implementation is not always easy (see Fig. 1).

Early neuroimaging studies exploited both internal and externally-generated stimuli. Initially, these experiments had block designs, where activity associated with each task (e.g. navigation in a VR environment, or recalling an autobiographical memory) was averaged across 30–60 second duration (e.g. Grön et al., 2000). Perhaps to the surprise of some, activations in predicted brain areas emerged, and where higher level control tasks were employed, focal activations, in for example the hippocampus, were also apparent (Maguire and Frith, 2003). Nevertheless, there were issues, not least of which was the control task. When dealing with highly complex stimuli, it was difficult to design an appropriate control task — in this situation what exact process does one control; how does one get the ‘level’ of the control task right? Even employing what was regarded by some as the lowest level of control, namely rest, proved problematic, not only because the contrast between rest and a very active task was interpretationally useless, but also given what emerged about the often highly active nature of rest (Stark and Squire, 2001), and the existence of the default mode (Mazoyer et al., 2001; Raichle et al., 2001; Shulman et al., 1997).

It became clear that for some experiments, the block design, while easiest to implement in the free-behaviour context, lacked fine-grained temporal resolution, and also reduced the correspondence to the real world, which is rarely organised in a blocked and orderly manner. Attempts to deal with this came in the form of experiments where the exposure to naturalistic stimuli, e.g. a large scale VR environment, occurred prior to scanning, while during scanning participants were exposed to particular aspects of the stimuli which were analysed as mini-blocks or in an event-related fashion (e.g. landmarks, VR characters they encountered; Aguirre and D'Esposito, 1997; Burgess et al., 2001; Janzen and Van Turennout, 2004). Others pursued a parametric approach by correlating aspects of performance (e.g. accuracy of navigation in a VR environment; Hartley et al., 2003; Peigneux et al., 2004; Addis et al., 2004) with the BOLD signal.

But the desire still persisted to capture real world interactions as they occurred and to derive temporal specificity for events within the unfolding interactive experience. This led to a range of further developments in the analytic techniques applied to fMRI data acquired during free-behaviour tasks; these included methods in three broad categories. First, where participants' classification of events (usually subsequent to scanning) was used to retrospectively analyse scanning data acquired during passive viewing, e.g. of movies (Zacks et al., 2001). Second, where participants' behaviour, content analysis, or subsequent verbal reports were used to analyse the fMRI data acquired during interactive tasks (Mathiak and Weber, 2006; Spiers and Maguire, 2006a). Third, stimulus-blind analyses, where ‘hidden’ patterns in brain activity were sought using mathematical algorithms such as independent components analysis (Calhoun et al., 2002; Malinen et al., 2007; now also available in model-free form – e.g. Schopf et al., 2011), reverse correlation procedures (Hasson et al., 2004), multi-voxel pattern analysis (MVPA; Haxby et al., 2001; Haynes and Rees, 2006; Norman et al., 2006), and decoding using receptive field models (Kay et al., 2008). The reader is referred to Spiers and Maguire (2007a) for a full discussion of these approaches and their advantages and disadvantages in the context of free-behaviour fMRI experiments. For illustrative purposes, and with blatant bias, here I describe our experience of executing such experiments that embodied some of the above methods.

## Some examples of it

I remember thinking about navigation experiments one morning in 2003 and subsequently calling my then post-doc Hugo Spiers to come and chat about this ‘bit of an idea’ that I had. The premise was simple, we get participants to navigate to destinations in a realistic VR town during fMRI. After the scan we play them back a video of their performance and ask them what they had been thinking during scanning, and use these retrospective verbal reports to analyse the fMRI time series; it seemed straightforward. However, months of research, pilot testing and technical manoeuvring, followed by gruelling training, scanning and debriefing of participants (see Fig. 1), and an 18 month data analysis tour de force meant this was the most ambitious fMRI study ever undertaken before or since in my laboratory.

Fortuitously, around this time a highly accurate and interactive VR simulation of central London, UK, became available, developed as the backdrop for commercial video game, enabling in situ navigation to be assessed in a controlled manner. ‘The Getaway’ (© Sony Computer Entertainment Europe) has over 110 km (70 miles) of London's driveable roads, accurately recreated from Ordinance Survey map data, covering fifty square kilometres (20 square miles) of the city centre. The one-way systems, working traffic lights, the busy London traffic, and an abundance of Londoners going about their business are all included (see snapshots in Fig. 1). Conveniently, one can simply navigate freely (with the game scenarios suspended) around the city using the game console, with a normal ground-level first person perspective, in a car of one's choice.

**Fig. 2:**
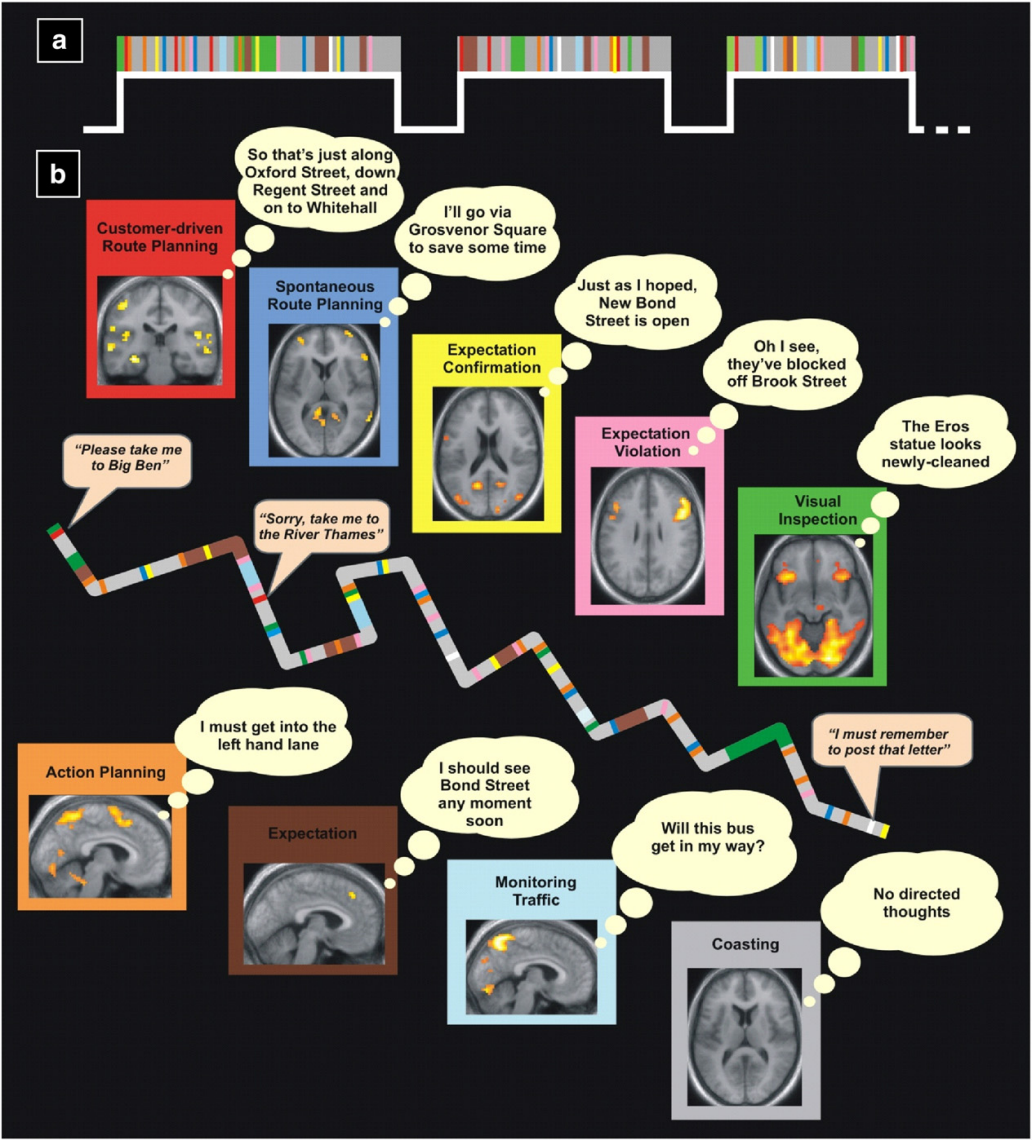
Neural correlates of navigation in VR London that resulted from the complex setup in Fig. 1 (Spiers and Maguire, 2006a). Panel (a) shows a schematic example of an fMRI time series after classification of a verbal report, with the time series segregated into different events and epochs corresponding to different categories of thought. Each participant's time series was unique. Panel (b) shows a cartoon of a route classified into a sequence of events and epochs of the different categories extracted from a verbal report. Speech callouts provide illustrative examples of auditory presentations of customers' requests and statements. On either side of the route are shown the results of the statistical comparison of the brain activity in each category with the baseline category ‘coasting’. Thought bubbles provide illustrative examples of thoughts described by participants. Examples of significantly active regions are shown overlaid on the mean structural scan of the 20 participants and include: *Customer-driven Route Planning*: left hippocampus; *Spontaneous Route Planning*: anterior prefrontal cortex, retrosplenial cortex; *Action Planning*: precuneus, pre-SMA, cerebellum; *Visual Inspection*: cuneus to parahippocampal cortex, anterior insula/ventrolateral prefrontal cortex; *Expectation Confirmation*: retrosplenial cortex, middle occipital gyrus; *Expectation Violation*: right lateral prefrontal cortex; *Expectation*: dorsomedial prefrontal cortex; *Monitoring Traffic*: precuneus; *Coasting*: no regions more active than the mean activity.

During fMRI, we had participants (who were licensed London taxi drivers) respond to customers' requests by delivering them to their required destinations in VR London, while driving a London taxi (Spiers and Maguire, 2006a). To gain an understanding of the navigation process on a second-by-second basis, immediately post-scan and without prior warning participants watched a video replay of their performance and were interviewed using a retrospective verbal report protocol. This involved getting participants to review their performance and report on what they had been thinking during the task in the scanner. Participants were able to produce detailed accounts of what they had been thinking during navigation, and were also clear about exactly when they had experienced particular thoughts. This enabled a complete specification of each participant's fMRI time series in terms of onsets and durations of event/epochs related to specific categories of thoughts (Fig. 2a) (12,484 events/epochs were identified overall). The precision of the timings was further tested using independent eye-tracking data acquired during the scan.

Analysis of the fMRI data revealed a complex choreography of neural responses comprising focal and distributed, transient and sustained brain activity, which fluctuated depending on circumstances and priorities (Fig. 2b). Not only did the study reveal the dynamic brain systems underpinning real world navigation to a degree that was not previously appreciated (see also Spiers and Maguire, 2008), but it provided new insights into the roles of specific brain areas. For example, the only change in hippocampal activity during the course of prolonged navigation was an increase during initial route planning. This was a striking revelation that provoked a re-think on our part about the role of the hippocampus, and fed into our subsequent ideas about a key function of the hippocampus being scene construction (Hassabis and Maguire, 2007, 2009; Hassabis et al., 2007b). This incredibly rich dataset also gave rise to novel findings about how the human brain processes proximity and distance to goal locations in an environment during navigation (Spiers and Maguire, 2007c), enabled a systematic characterisation of the neural substrates of driving behaviour in a real city (Spiers and Maguire, 2007b), and provided new insights into how the brain supports spontaneous mentalising during real-world experiences (Spiers and Maguire, 2006b).

But it is not only in relation to navigation in large-scale space that naturalistic fMRI studies make potentially important contributions. Perhaps one of the ultimate ecological challenges is to take each participant's individual, complex and unique autobiographical memories, and devise a means to understand how they are instantiated in the brain, and how they are influenced by time, age and pathology. This has been of long-standing interest in neuroscience (Scoville and Milner, 1957) and, as with navigation, fMRI has revealed a distributed set of brain regions supporting autobiographical memory (Maguire, 2001; Svoboda et al., 2006). Indeed fMRI allowed us to appreciate that the brain network that supports navigation is highly overlapping with that underpinning autobiographical memory and also imagination of the future, an observation that continues to generate significant theoretical interest (Buckner and Carroll, 2007; Hassabis and Maguire, 2007, 2009; Schacter and Addis, 2007; Spreng et al., 2009).

A relatively recent development in fMRI data analysis offers further opportunities for moving our understanding of autobiographical memory forward. Facilitated by high spatial resolution fMRI (Carr et al., 2010), multi-voxel pattern analysis using support vector machine linear classifiers takes into account the patterns of fMRI BOLD response across multiple voxels, the idea being that important findings could be missed from conventional analysis of fMRI data (which focus on responses in individual voxels) if information is represented in distinct patterns across voxels rather than in the number of separate voxels that reach a threshold of activation. If a classifier is successful at predicting the correct stimulus solely from the patterns of fMRI BOLD, it must mean that there is information about that stimulus represented in the brain region where the pattern of voxels was identified. This ‘decoding’ approach is interesting, therefore, not only because it reveals pattern information that eludes conventional fMRI studies, but one can examine these patterns in individual participants, and most crucially, look at patterns of fMRI activity associated with specific stimuli or specific memories.

**Fig. 3:**
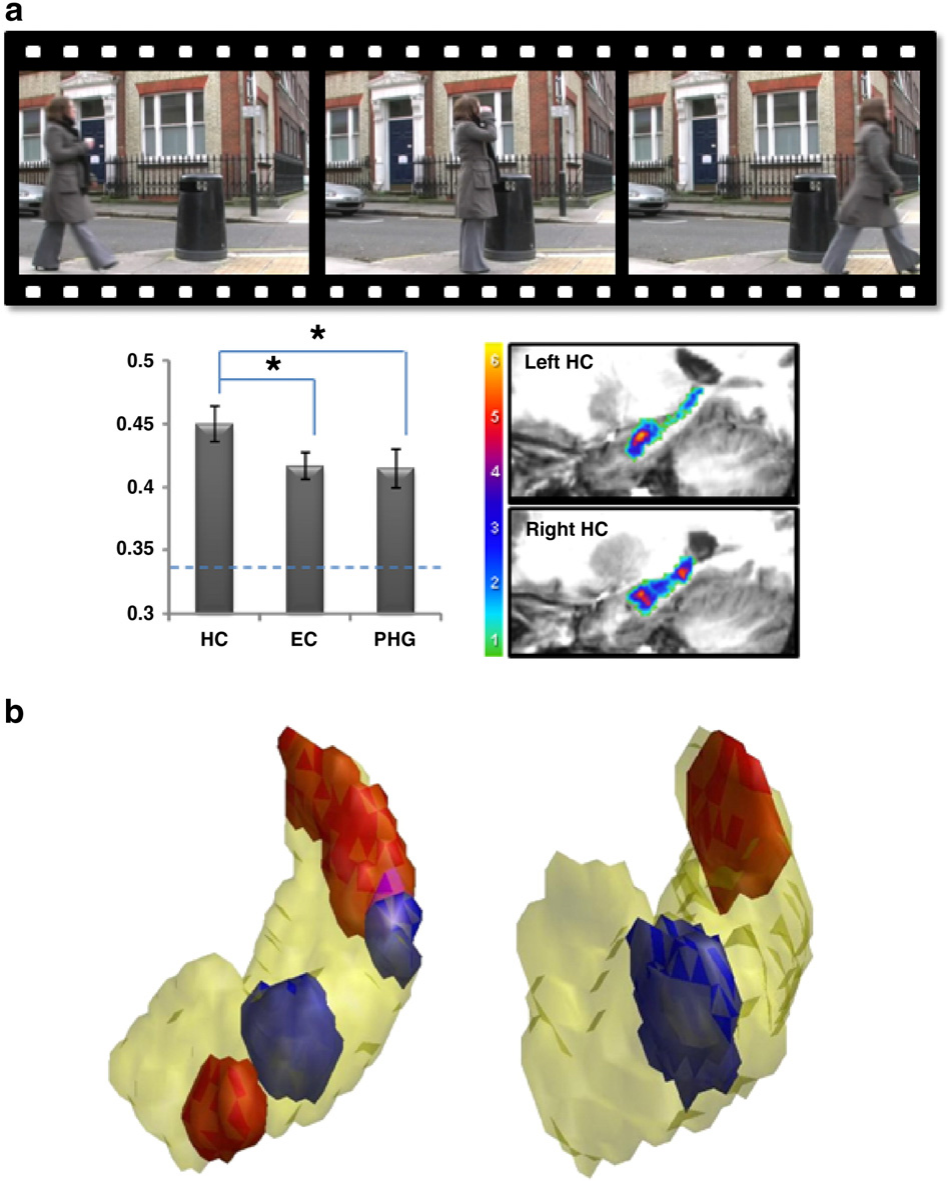
The combination of naturalistic stimuli and multi-voxel pattern analysis. Panel (a): participants saw short movie clips prior to scanning and then had to recall them during fMRI (Chadwick et al., 2010). Using MVPA it was possible to predict which movie was being recalled from patterns of fMRI BOLD across voxels in the hippocampus (HC), moreso than from neighbouring medial temporal lobe regions, entorhinal cortex (EC) and parahippocampal gyrus (PHG), which we interpret as implying the presence of episodic-like memory traces in the hippocampus. Panel (b): using MVPA is it possible to re-project key discriminating voxels back into the brain to examine the location and distribution of information (information maps). In this example, the information maps pertaining to two different types of memory, in red and blue, are shown for two example 3-D rendered hippocampi. The minimal overlap (pink) between the information maps is clear, and indicates that MVPA can be used to inform possible intra-hippocampal regional distinctions, for example here between anterior and posterior portions.

Using this approach we found that patterns of activity across voxels in the hippocampus could be used to predict which of three movie clips was being recalled (Fig. 3a; Chadwick et al., 2010). We interpreted this to imply that memory representations (or ‘traces’) of the movies were present in the hippocampus, with more information about the memories available in the hippocampus compared with adjacent medial temporal lobe regions such as entorhinal and parahippocampal cortices.

We have since moved on to consider what specific aspects of episodic memories can be decoded by MVPA. Using green-screen technology we created four highly-overlapping movies of everyday events (Chadwick et al., 2011). Participants were scanned using high-resolution fMRI whilst recalling the movies. MVPA revealed that the hippocampus supported distinct representations of each memory, while neighbouring regions did not, demonstrating that the human hippocampus maintains unique pattern-separated memory traces even when memories are highly overlapping. The hippocampus also contained representations of spatial contexts that were shared across different memories, consistent with a specialised role in processing space (O’Keefe and Nadel, 1978). Together, this set of findings suggests that the hippocampus is capable of supporting at least two different types of representation — each memory has a unique representation created through a process of pattern separation, and at the same time spatial backdrops that are common to different memories are also represented in the hippocampus.

One of the key debates in memory neuroscience concerns the issue of system-level consolidation and the timescale of hippocampal involvement in representing episodic/autobiographical memories. Consolidation Theory suggests that once consolidated, autobiographical memories are no longer represented in the hippocampus and instead become neocortically-dependent (Squire, 1992). By contrast others (e.g. Multiple Trace Theory — Nadel and Moscovitch, 1997; Scene Construction theory — Hassabis and Maguire, 2007) posit that the hippocampus is necessary for supporting rich and vivid autobiographical memories whether recent or remote. Given that MVPA permits us to examine individual memory representations (e.g. Chadwick et al., 2010, 2011), it may be possible to use MVPA to explore recent and remote autobiographical memories, offering new leverage on this long-standing and controversial issue. For example, might it be possible to decode remote autobiographical memories from patterns of activity in the hippocampus, or have such memories been consolidated out of the hippocampus and so no longer leave a detectable trace there? Thus, the combination of MVPA and naturalistic stimuli such as autobiographical memories may open up a range of new opportunities to make genuine conceptual advances.

## Conclusions

Here, briefly, and in a highly subjective and selective manner, I considered the study of the freely-behaving brain using fMRI. I conclude by noting that it is not always necessary to employ a naturalistic approach, it is not better by default, and to deploy ecological paradigms properly can be very challenging. Is it worth it — yes, undoubtedly. The judicious use of naturalistic stimuli and designs, where key neuroscience questions cannot be addressed effectively in other ways, is an invaluable addition to the cognitive neuroscience armamentarium. From my perspective, coming from a background in clinical neuropsychology, and seeing patients struggle with the everyday tasks of navigating in familiar places and trying to maintain some sense of their personal past, the opportunity to look directly at the neural instantiation of such behaviours in vivo using fMRI is still a wondrous thing. Early free-behaviour designs were simple, and in some sense the findings were largely confirmatory. However, the increasing sophistication of stimuli and in particular innovations in data analysis mean that naturalistic fMRI studies are now able to contribute novel insights to key theoretical debates. In short, the future is bright for keeping it real.
